# Characterization of CD4+ T cells primed and boosted by MHCII primary uveal melanoma cell-based vaccines

**DOI:** 10.18632/oncotarget.26737

**Published:** 2019-03-05

**Authors:** Julia M. Kittler, Jonas Sommer, Anika Fischer, Sabine Britting, Margarete M. Karg, Barbara Bock, Imke Atreya, Ludwig M. Heindl, Andreas Mackensen, Jacobus J. Bosch

**Affiliations:** ^1^ Department of Internal Medicine 5 – Hematology and Oncology, University Hospital Erlangen, Friedrich-Alexander-University Erlangen-Nuremberg (FAU), Erlangen, Germany; ^2^ Department of Internal Medicine 1 – Gastroenterology, Pneumology and Endocrinology, University Hospital Erlangen, Friedrich-Alexander-University Erlangen-Nuremberg (FAU), Erlangen, Germany; ^3^ Department of Ophthalmology and Center for Integrated Oncology (CIO) Cologne-Bonn, University of Cologne, Cologne, Germany

**Keywords:** uveal melanoma, tumor immunity, T cell activation, CD4+ T cells, ICAM-1

## Abstract

Uveal melanoma is the most common primary malignancy of the eye in adults. Despite significant improvements in treatment of the primary tumor, to date none of these therapies prevent metastatic disease or improve overall survival. We are exploring immunotherapeutic options for metastatic uveal melanoma using MHC II uveal melanoma cell-based vaccines that target the activation of tumor-reactive CD4+ T cells. Previously, we showed that these uveal melanoma cell-based vaccines activate CD4+ T cells within total peripheral blood lymphocytes (PBMC). Since PBMC include professional antigen presenting cells, we now demonstrate that Mel202/DR1/CD80 vaccine cells directly activate a diverse repertoire of purified, naïve CD4+ T cells. The activated CD4+ T cells proliferated, secreted high amounts of interferon gamma (IFNγ) and produced a heterogeneous profile of Th1, Th2 and Th17 cytokines. Analysis of the TCR-Vβ-repertoire showed that a polyclonal T cell response was induced, suggesting the capacity of vaccine-activated CD4+ T cells to target multiple tumor (neo)antigens. In addition, a subset of the responding CD4+ T cells expressed forkhead box protein P3 (FoxP3), indicating that although a regulatory component of the vaccine-activated CD4+ T cell response was induced, the anti-tumor vaccine response was not limited by these regulatory CD4+ T cells. Finally, Mel202/DR1/CD80 uveal melanoma vaccine cells expressed the intercellular adhesion molecule 1 (ICAM-1) that was pivotal for CD4+ T cell activation via lymphocyte function-associated antigen 1(LFA-1). In conclusion, MHC II uveal melanoma vaccines activate purified CD4+ T cells and may serve as a novel immunotherapy for uveal melanoma patients.

## INTRODUCTION

Uveal melanoma is an aggressive primary malignancy of the eye. There have been significant improvements in treatment of the primary tumor. Although enucleation of the tumor-bearing eye is still an option, a variety of sight-saving treatments are available e.g. local radiotherapy, proton-beam irradiation or trans-scleral resection [1]. The challenge is that none of these therapies prevent metastatic spread, which develops in ∼50% of patients with large tumors [2–4]. Metastases occur primarily in the liver (93%), but also in the lung (24-55%) and other visceral organs [5]. Metastatic patients have been treated with conventional chemotherapies, novel targeted therapies and immunotherapy by checkpoint blockade. Unfortunately, none of these modalities, including checkpoint blockade, have improved overall survival. The need for novel therapies is evident, particularly since significant progress in the treatment of metastatic cutaneous melanoma has been made in recent years.

We are exploring immunotherapeutic options for metastatic uveal melanoma. Specifically, the option of adoptive cell transfer (ACT) with tumor-specific T cells may be a promising therapeutic strategy. In ACT, tumor-infiltrating or circulating autologous lymphocytes can be isolated, *in vitro* selected, expanded and then reinfused into the patient. Numerous trials in patients with cutaneous metastatic melanoma have been undertaken to prove the feasibility and efficacy of this approach (reviewed in [6]). In general, the challenge is to obtain sufficient numbers of tumor-specific T cells for ACT. We hypothesize that tumor cell-based vaccines can facilitate the acquisition of tumor-specific T cells *ex vivo*. ACT with tumor-specific T cells may be particularly suitable for uveal melanoma, since the primary tumor originates in the immune privileged environment of the eye and may process and present tumor (neo)antigens to which the host’s own immune system is not tolerized. Previously, we have generated cell-based vaccines that consist of primary uveal melanoma cells genetically modified to express major histocompatibility class II (MHC II) alleles syngeneic to the recipient and the costimulatory molecule CD80 (B7.1). The vaccine cells were able to activate CD4+ T cells that react with primary uveal melanoma cells and cross-react with metastatic uveal melanoma cells [7]. In addition, cytotoxic CD8+ T cells (CTL) were activated to become cytolytic towards primary and metastatic uveal melanoma cells [8]. Moreover, the expression of CD80 blocked the interferon gamma (IFNγ)-mediated upregulation of programmed-death-ligand 1 (PD-L1) and thereby prevented T cell suppression during vaccine priming and boosting of responding T cells [9]. The MHC II vaccine concept was further validated *in vitro* in human breast and lung carcinoma models [10–12]. Furthermore, MHC II vaccines made from murine sarcoma, mammary carcinoma and melanoma cells activated tumor-specific CD4+ T cells and mediated rejection of established primary and metastatic mouse tumors, validating the MHC II vaccine concept *in vivo* in animal models [13–16].

Activation of CD4+ T lymphocytes is the main goal of our vaccine strategy. CD4+ T cells are pivotal for CD8+ T cell-mediated immunity [17], either through their function as “helper” T cells that provide cytokine support for CD8+ T cells [18, 19] or by their induction of CD40 expression on dendritic cells (DC) (“licensing”), which in turn activate CD8+ T cells [20–22]. CD4+ T cells are also essential for generating CD8+ T memory cells and for preventing tolerance induction of CD8+ T cells [23, 24]. In addition, IFNγ production by effector CD4+ T cells facilitates anti-tumor reactivity by blocking neo-vascularization, directly inhibiting tumor cell proliferation and upregulating tumor-expressed MHC molecules that improve CTL recognition [25]. CD4+ T cells can also become directly cytolytic to tumor cells [26], for example via tumor necrosis factor (TNF)-related apoptosis-inducing ligand (TRAIL) [27] or Fas/Fas ligand (FasL) pathways [28].

In our previous studies, MHCII vaccines activated CD4+ T cells in the context of total peripheral blood lymphocytes (PBMC). In the present study, we showed the capability of the Mel202/DR1/CD80 vaccine cells to directly prime and boost a diverse repertoire of highly purified, naïve CD4+ T cells isolated from PBMC. The activated CD4+ T cells expressed activation markers, proliferated, secreted high amounts of IFNγ and produced a heterogeneous profile of T helper type 1 (Th) 1, Th2 and Th17 cytokines. Analysis of the T cell receptor (TCR)-Vβ-repertoire revealed that a polyclonal, diverse CD4+ T cell response was induced, suggesting the capacity of vaccine-activated CD4+ T cells to target multiple tumor (neo)antigens. Mel202/DR1/CD80 vaccine cells expressed the intercellular adhesion molecule 1 (ICAM-1; CD54) that was required for CD4+ T cell activation via lymphocyte function-associated antigen 1 1 (LFA-1; CD11a). Although a subset of the activated CD4+ T cells expressed forkhead box protein P3 (FoxP3) and appeared to be T regulatory cells (Tregs), these cells did not significantly impact the anti-tumor vaccine response.

## RESULTS

### Mel202/DR1/CD80 uveal melanoma vaccines prime and boost purified CD4+ T cells

**Figure 1:**
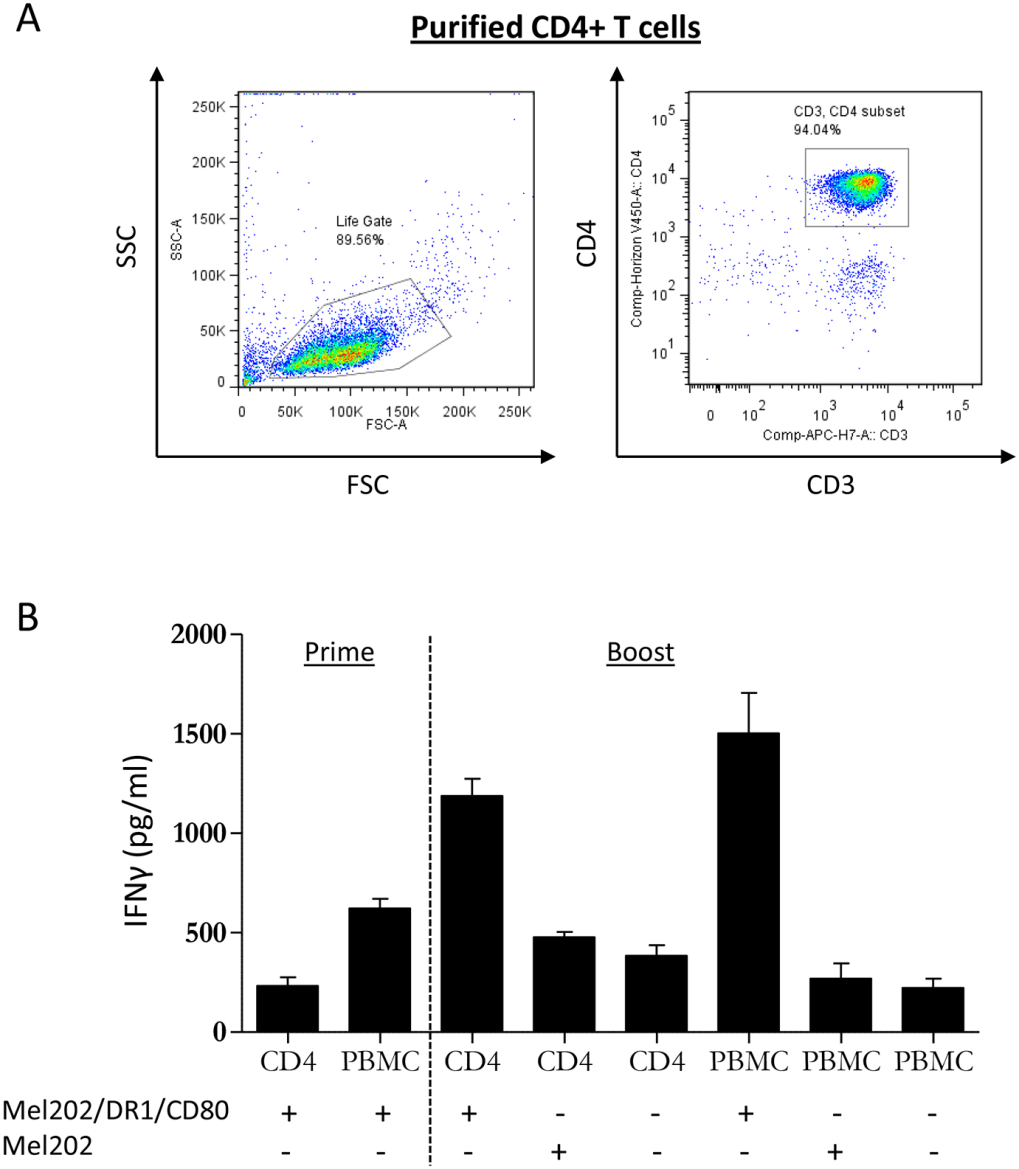
MHC II vaccines prepared from primary uveal melanoma cells prime and boost PBMC and purified CD4+ T cells **(A)** Starting population of purified CD4+ T cells at the start of the experiment. CD3+/CD4+ T cells were analyzed within the live gate by flow cytometry. **(B)** PBMC or purified CD4+ T cells from HLA-DR1 positive donors were primed with Mel202/DR1/CD80 and boosted with Mel202/DR1/CD80 or parental Mel202 cells. Purity of CD4+ T cells was >90% throughout the course of the experiments. T cell activation was quantified by measuring IFNγ-release. Data are representative of three independent experiments with PBMC or purified CD4+ T cells from 3 individual donors.

To investigate whether Mel202/DR1/CD80 vaccine cells are capable of directly activating purified CD4+ T cells, we first isolated naïve CD4+ T cells from PBMC of healthy human leukocyte antigen (HLA)-DR1+ donors (Figure 1A). Subsequently, PBMC or purified CD4+ T cells were co-cultured with irradiated Mel202/DR1/CD80 vaccine cells. Controls included Mel202 wild type cells or T cells alone. After priming and boosting, purified CD4+ T cells that had been co-cultured with Mel202/DR1/CD80 vaccine cells produced IFNγ at a concentration comparable to the IFNγ production by PBMC (Figure 1B). These data demonstrate that CD4+ T cells are directly activated by uveal melanoma vaccines.

### Mel202/DR1/CD80 vaccine cells activate CD4+ T cells that secrete IFNγ and produce a heterogeneous profile of Th1/Th2 cytokines

**Figure 2:**
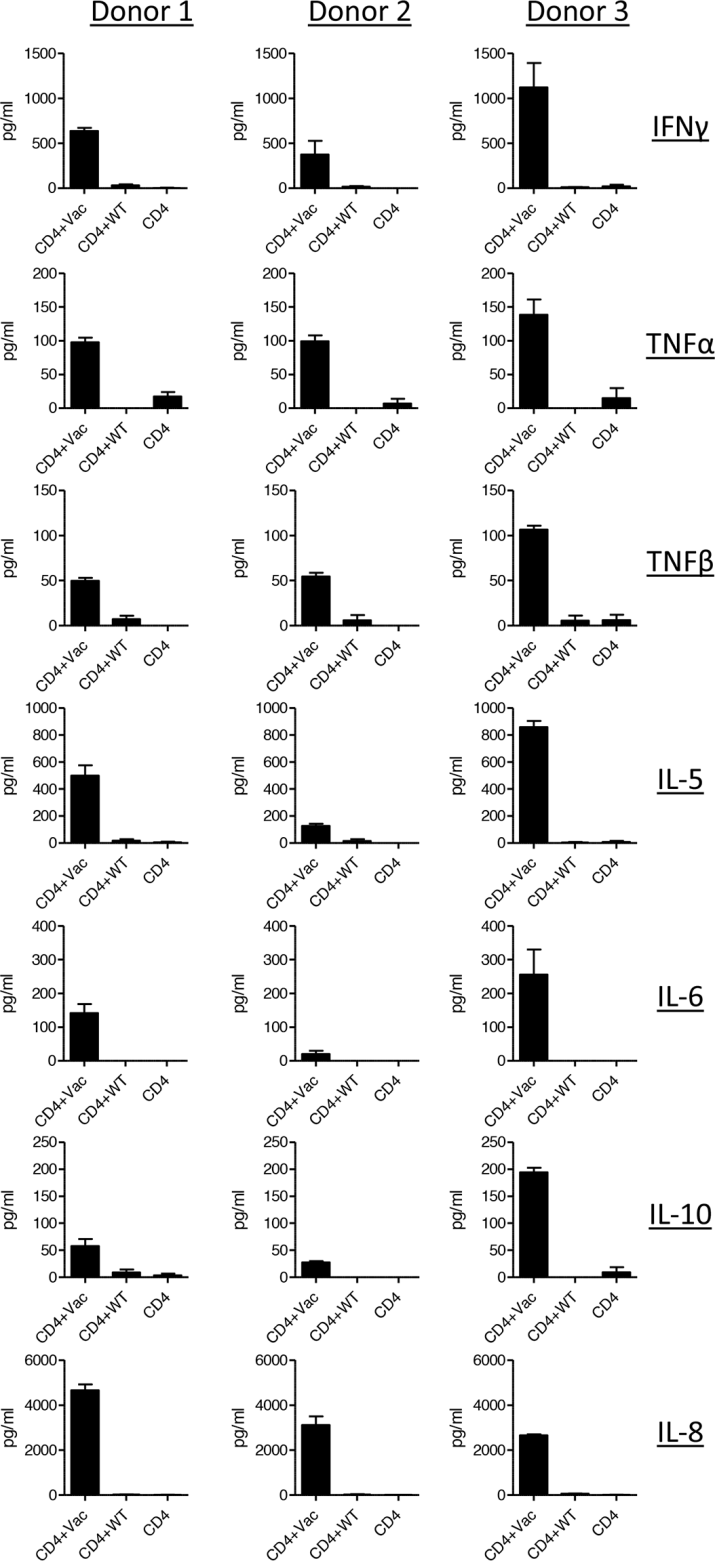
Vaccine-activated CD4+ T cells secrete various Th1 and Th2 type cytokines Purified CD4+ T cells from HLA-DR1 positive donors were primed with Mel202/DR1/CD80 and boosted with Mel202/DR1/CD80 (CD4 + vac) or parental Mel202 (CD4 + wt) cells. Th1 (IFNγ, TNFα and TNFβ) and Th2 type (IL-5, IL-6, IL-8, IL-10) cytokine release in the supernatant was measured by a flowcytometric multiplex bead array. Purity of CD4+ T cells was >90% throughout the course of the experiments. Data are pooled from three independent experiments with purified CD4+ T cells for each donor 1, 2 or 3.

Different subsets of CD4+ T helper cells are defined by their individual cytokine profile. As the distinctive T helper cell subsets are linked with different functions regarding activation or suppression of the immune response, we addressed the question which kind of T helper cell subsets are activated by Mel202/DR1/CD80 vaccines. The analysis of a broad spectrum of cytokines secreted upon boosting showed that the Mel202/DR1/CD80 vaccine-activated CD4+ T cells produce a heterogeneous profile of cytokines, typical for both Th1 and Th2 cells. Regarding Th1 cytokines, the vaccine-activated CD4+ T cells secreted IFNγ, TNFα and TNFβ (Figure 2). No production of interleukin (IL)-12 (p70) was detected (data not shown). As for Th2 cytokines, the vaccine-activated CD4+ T cells secreted IL-5, IL-6 and IL-10 (Figure 2). No production of IL-4 or IL-1β was detected (data not shown). Vaccine-activated CD4+ T cells also produced IL-8 (Figure 2). Cytokine production varied between the three different donors. In summary, Mel202/DR1/CD80 vaccine-activated CD4+ T cells secrete various Th1 and Th2 cytokines, suggesting different subsets of CD4+ Th cells may be activated.

### Mel202/DR1/CD80 vaccine-activated CD4+ T cells proliferate

**Figure 3:**
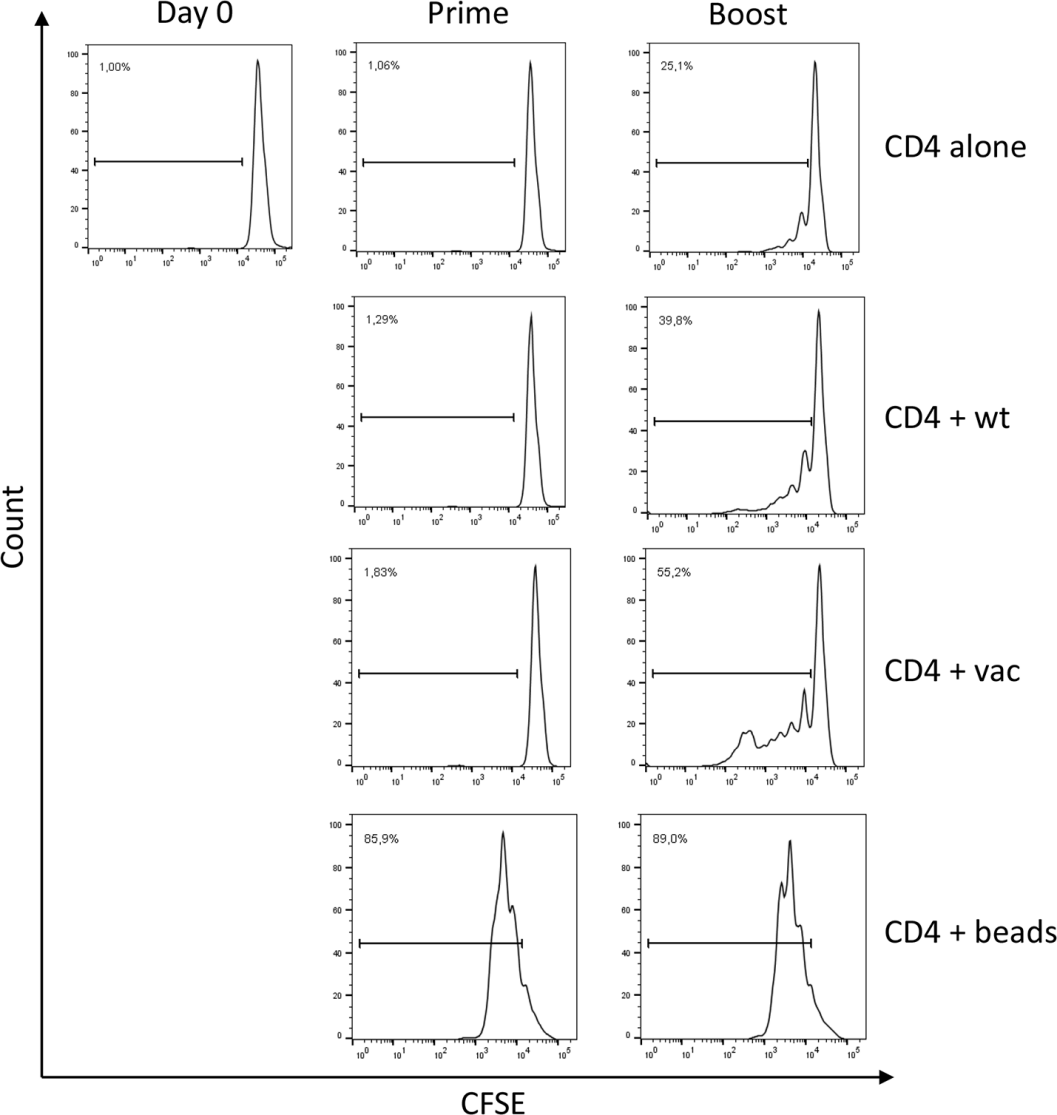
Vaccine-activated CD4+ T cells proliferate Purified CD4+ T cells were CFSE labeled, primed with Mel202/DR1/CD80 and boosted with Mel202/DR1/CD80 (CD4 + vac) or parental Mel202 (CD4 + wt) cells. Purified CD4+ T cells stimulated with anti-CD3/CD28 beads (CD4 + beads) or purified CD4+ T cells without stimulation (CD4 alone) were used as positive and negative controls respectively. CFSE labeled CD4+ T cells were analyzed after gating on CD3+/CD4+ T cells within the live gate. Proliferation was quantified by measuring the number of CD4+ T cells within the dividing gate. Percentages indicate % dividing of total CD4+ T cells. Purity of CD4+ T cells was >90% throughout the course of the experiments. Data are representative of three independent experiments with purified CD4+ T cells from 3 individual donors.

To further verify that Mel202/DR1/CD80 primed and boosted CD4+ T cells were activated, CD4+ T cells were analyzed for proliferation. As indicated by CFSE dilution, there is no proliferation detected after priming. However, after boosting, a 1.4 – 2.2 fold increase of proliferative activity as compared to the controls was detected (Figure 3). Only a 1.6 fold lower proliferative activity was detected as compared to standard CD3/CD28 bead stimulation. In conclusion, priming and boosting with Mel202/DR1/CD80 vaccines leads to proliferation of CD4+ T cells.

### Mel202/DR1/CD80 vaccine cells induce a polyclonal CD4+ T cell response

**Figure 4:**
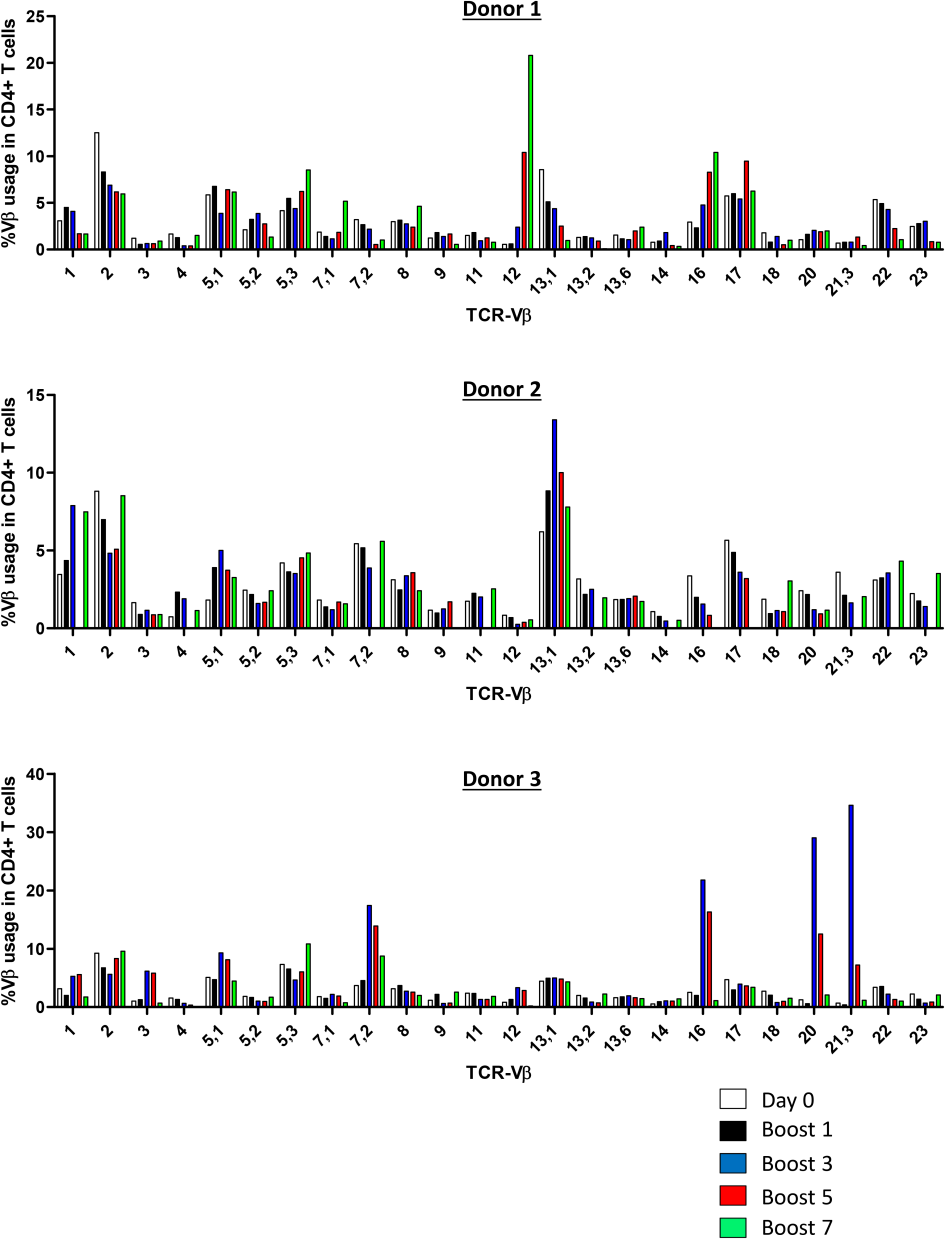
Vaccine-activated CD4+ T cells display a diverse polyclonal TCR-Vβ repertoire Purified CD4+ T cells were primed and repetitively boosted (7x) with Mel202/DR1/CD80 vaccine cells. CD3+/CD4+ T cells were gated and analyzed by flow cytometry for TCR-Vβ usage on day 0 and after boosts 1, 3, 5, and 7. Each colored bar denotes the different time points of analysis. Purified CD4+ T cells from 3 individual donors were used. Purity of CD4+ T cells was >90% throughout the course of the experiments. Data are representative of two independent experiments for each donor 1, 2, and 3.

To orchestrate an efficient anti-tumor immune response to uveal melanoma cells, it is necessary to activate and expand T cells with a TCR specific to uveal melanoma (neo)antigens. Uveal melanoma vaccine cells potentially present a diverse repertoire of tumor (neo)antigens restricted to HLA-DR and may therefore activate a polyclonal CD4+ T cell response. Since the antigens presented by the uveal melanoma vaccine cells are unknown, we determined TCR-Vβ usage of vaccine-activated CD4+ T cells upon repetitive stimulation with Mel202/DR1/CD80 vaccines. At day 0, TCR-Vβ expression corresponded with the distribution in the standard Caucasian population (Figure 4). Upon an increasing number of restimulations (up to 7 boosts), a polyclonal CD4+ T cell response was detected (Figure 4). Expression of some individual TCR-Vβs was enriched, in particular Vβ5.1, Vβ7.2, Vβ12, Vβ13.1 and Vβ16. As expected, donor dependent variation of TCR-Vβ usage was observed. In summary, the induction of a polyclonal CD4+ T cell response indicates activation of a diverse repertoire of CD4+ T cells and suggests T cell reactivity against multiple tumor (neo)antigens.

### A diverse CD4+ T cell response is maintained by repetitive boosting with Mel202/DR1/CD80 vaccines

**Figure 5:**
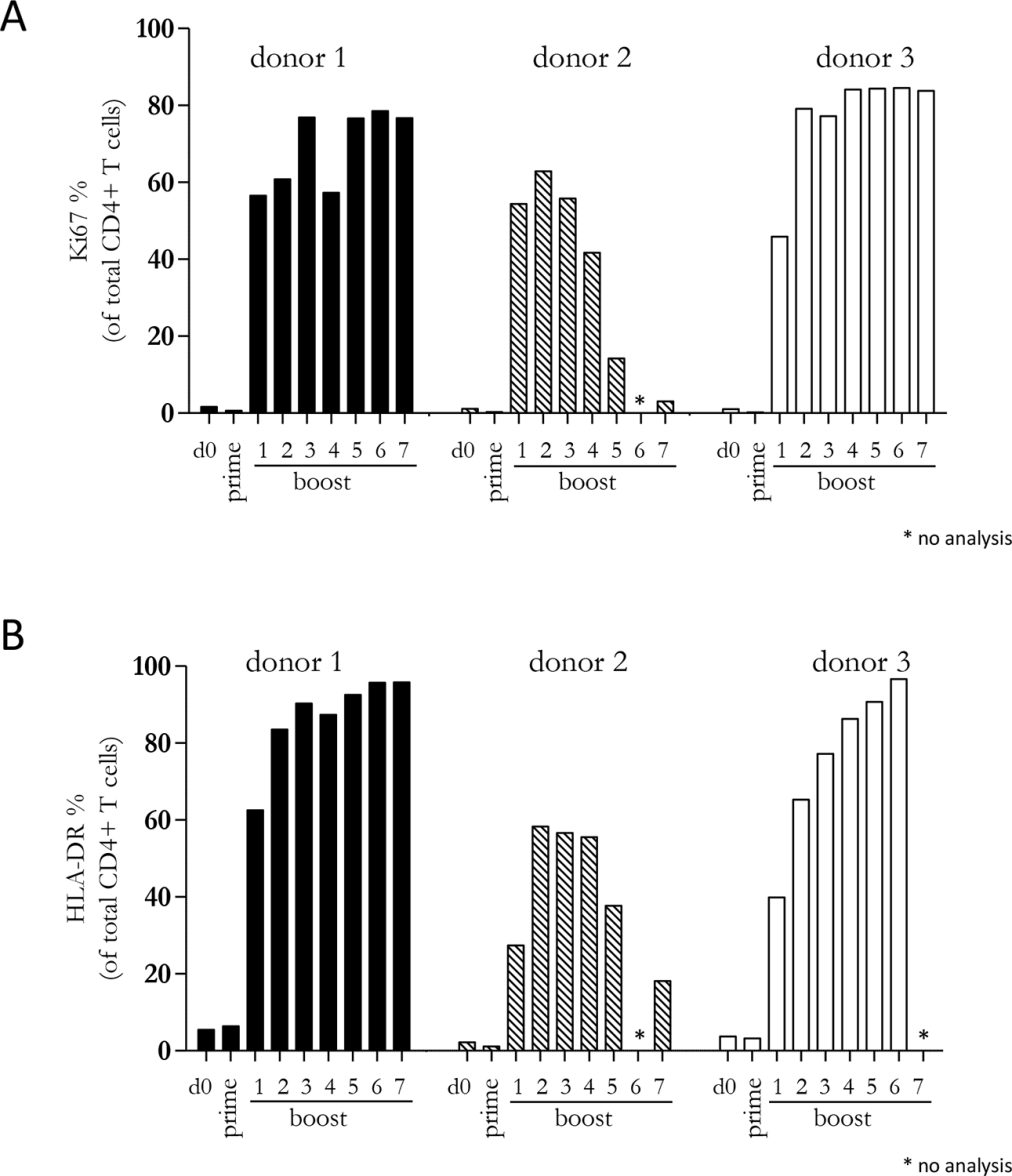
Vaccine-activated CD4+ T cells maintain expression of proliferation and activation markers Purified CD4+ T cells were primed and repetitively boosted (7x) with Mel202/DR1/CD80 vaccine cells. CD3+/CD4+ T cells were gated and analyzed by flow cytometry for expression of **(A)** Ki67 and **(B)** HLA-DR on day 0, after prime and boosts 1-7. Purity of CD4+ T cells was >90% throughout the course of the experiments. Data are representative of two independent experiments with purified CD4+ T cells for each donor 1, 2, and 3.

**Figure 6:**
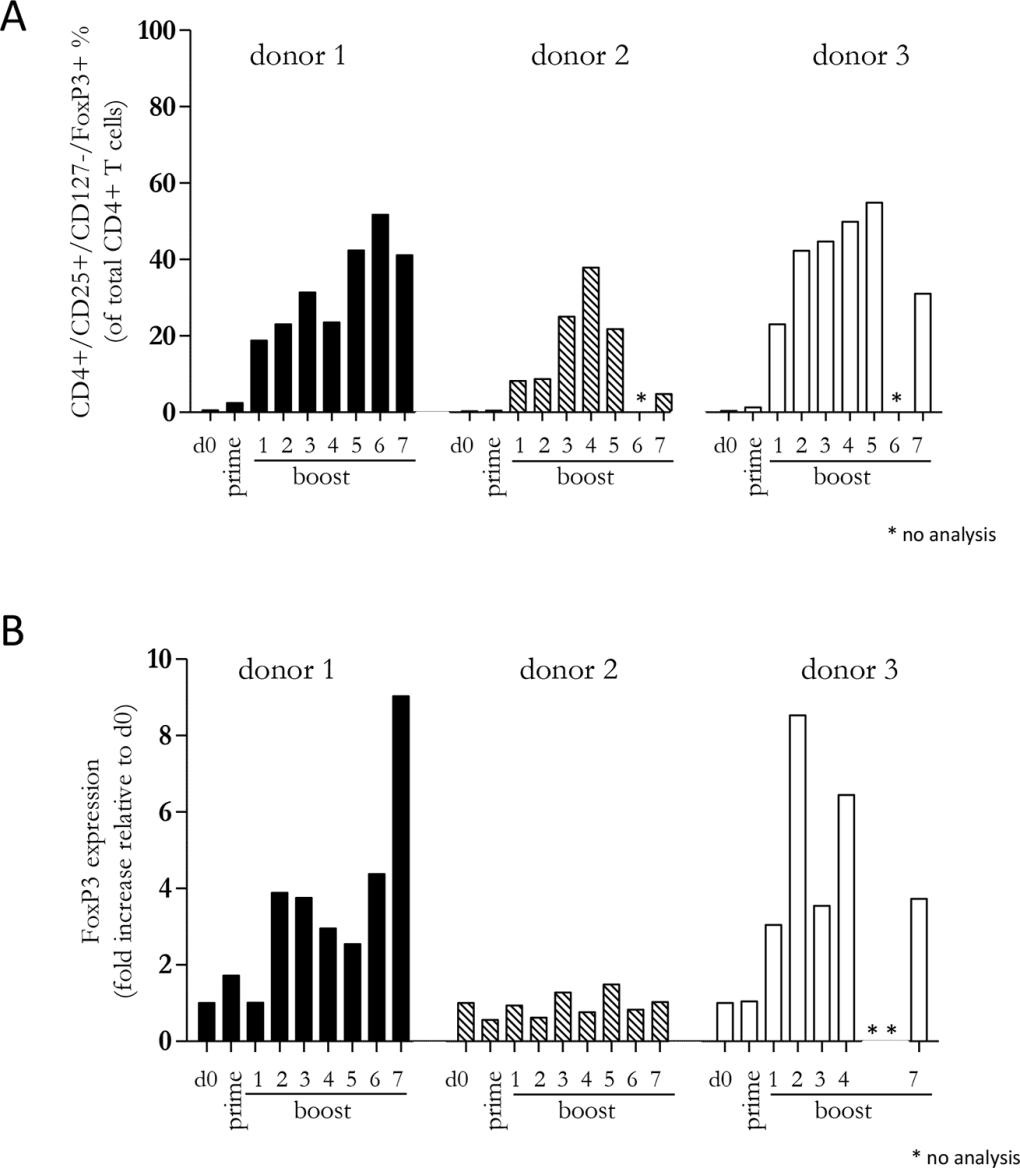
Vaccine-activated CD4+ T cells include a subpopulation with a regulatory phenotype Purified CD4+ T cells were primed and repetitively boosted (7x) with Mel202/DR1/CD80 vaccine cells. **(A)** CD3+/CD4+ T cells were gated and analyzed by flow cytometry for expression of CD25, CD127 and FoxP3 on day 0, after prime and boosts 1-7. **(B)** FoxP3 gene expression was analyzed by qRT-PCR isolated from the total pool of vaccine-activated CD4+ T cells on day 0, after prime and boosts 1-7. Purity of CD4+ T cells was >90% throughout the course of the experiments. Data are representative of two independent experiments with purified CD4+ T cells for each donor 1, 2, and 3.

**Figure 7:**
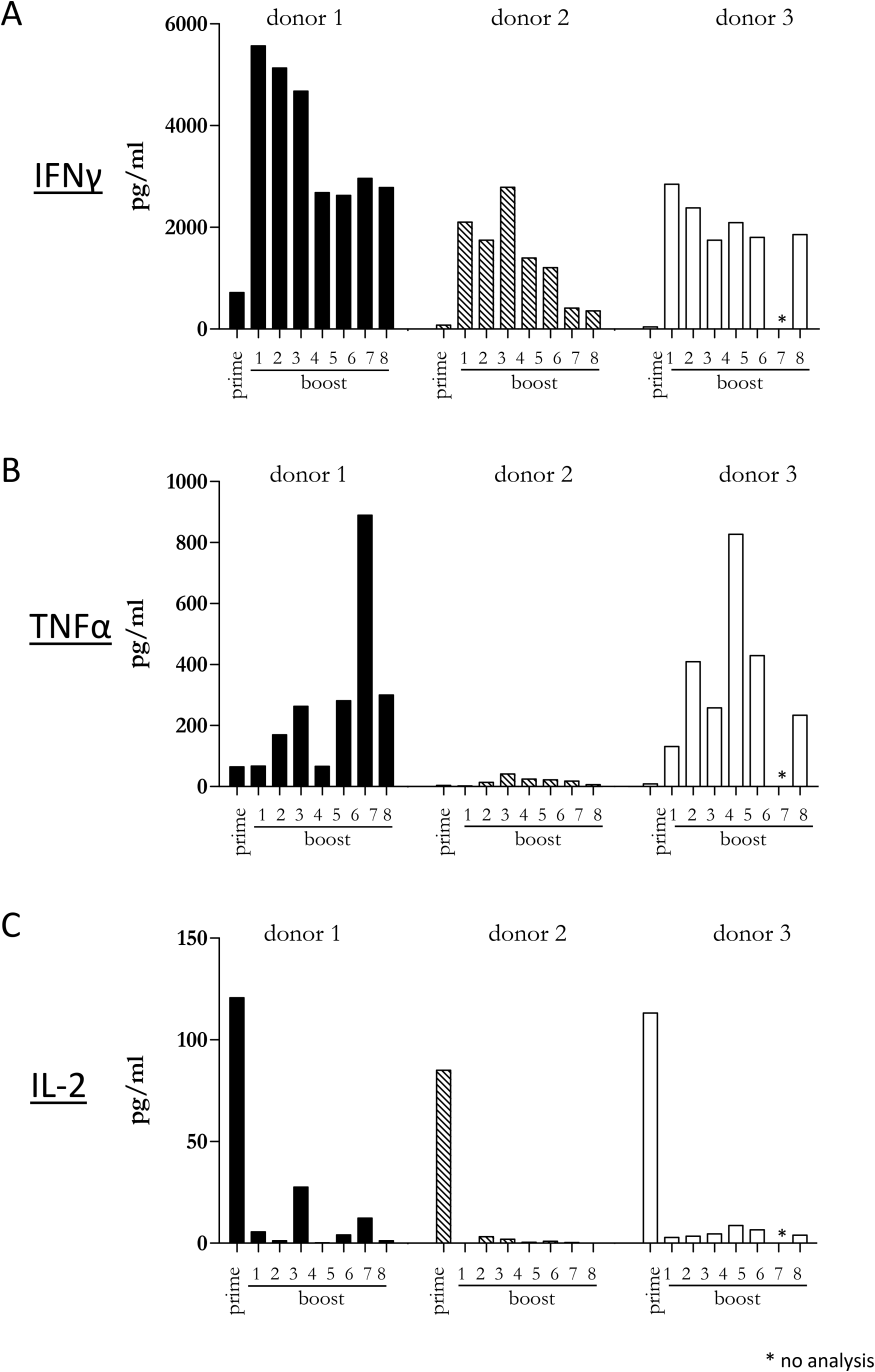
Vaccine-activated CD4+ T cells maintain Th1 cytokine production and secrete IL-2 at priming Purified CD4+ T cells were primed and repetitively boosted (8x) with Mel202/DR1/CD80 vaccine cells. **(A)** IFNγ, **(B)** TNFα and **(C)** IL-2-release in the supernatant was measured by a flowcytometric multiplex bead array after prime and boosts 1-8. Purity of CD4+ T cells was >90% throughout the course of the experiments. Data are representative of two independent experiments with purified CD4+ T cells for each donor 1, 2, and 3.

**Figure 8:**
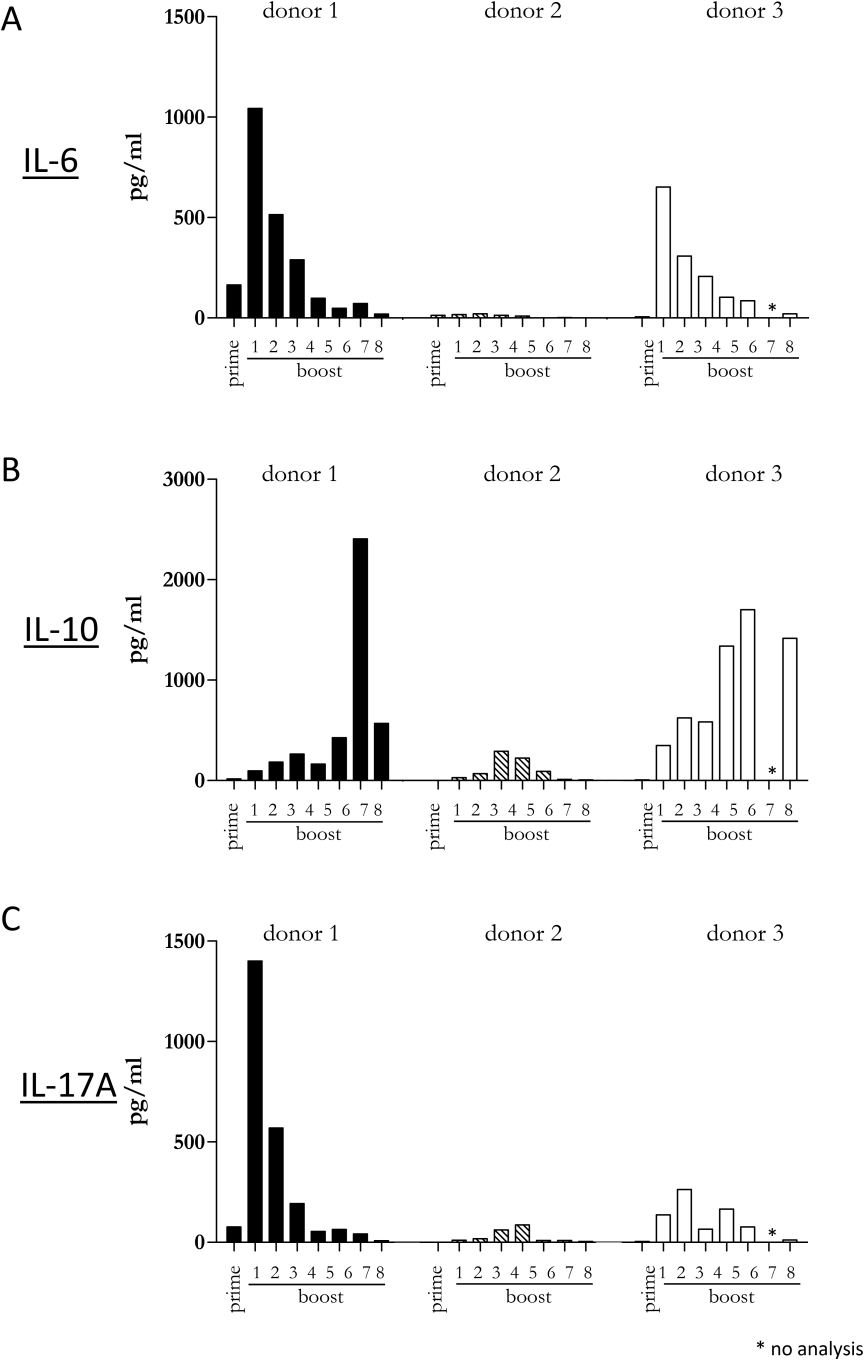
Vaccine-activated CD4+ T cells maintain Th2 and Th17 cytokine production Purified CD4+ T cells were primed and repetitively boosted (8x) with Mel202/DR1/CD80 vaccine cells. **(A)** IL-6, **(B)** IL-10 and **(C)** IL-17A-release in the supernatant was measured by a flow cytometric multiplex bead array after prime and boosts 1-8. Purity of CD4+ T cells was >90% throughout the course of the experiments. Data are representative of two independent experiments with purified CD4+ T cells for each donor 1, 2, and 3.

Since our previous experiments demonstrated that a diverse repertoire of CD4+ T cells is induced by Mel202/DR1/CD80 vaccines, we next determined whether these vaccine-activated CD4+ T cells maintained their activation status. Repetitive *in vitro* boosting by Mel202/DR1/CD80 vaccines maintained the proliferative activity of the responding CD4+ T cells as determined by the expression of the proliferation marker Ki67 (Figure 5A). In addition, the repetitively boosted CD4+ T cells continued to express the activation marker HLA-DR (Figure 5B). CD4+ T cells also contain a population of Tregs, which can be defined by CD25 and FoxP3 expression, and absence of CD127 expression [29]. A subset of Mel202/DR1/CD80 vaccine-activated CD4+ T cells expressed this regulatory phenotype (CD4+/CD25+/CD127-/FoxP3+) (Figure 6A) and upregulated FoxP3 gene expression (Figure 6B), indicating that although a regulatory component of the vaccine-activated CD4+ T cell response was induced, the anti-tumor response marked by IFNγ-release and CD4+ T cell proliferation was not limited by these regulatory CD4+ T cells. Mel202/DR1/CD80 vaccine-activated CD4+ T cells continued to secrete IFNγ (Figure 7A) and various Th1 (Figure 7B-7C), Th2 (Figure 8A-8B) and Th17 (Figure 8C) cytokines upon repetitive boosting with Mel202/DR1/CD80 vaccines. Of note, IL-2 was primarily secreted after priming (Figure 7C). Donor dependent variation was observed indicating variable vaccine-responsiveness of CD4+ T cells between donors. Collectively, our data indicate that Mel202/DR1/CD80 uveal melanoma vaccine cells maintain the activation of various subtypes of CD4+ T cells.

### The ICAM-1/LFA-1 interaction is required for activation of CD4+ T cells by Mel202/DR1/CD80 vaccines

**Figure 9:**
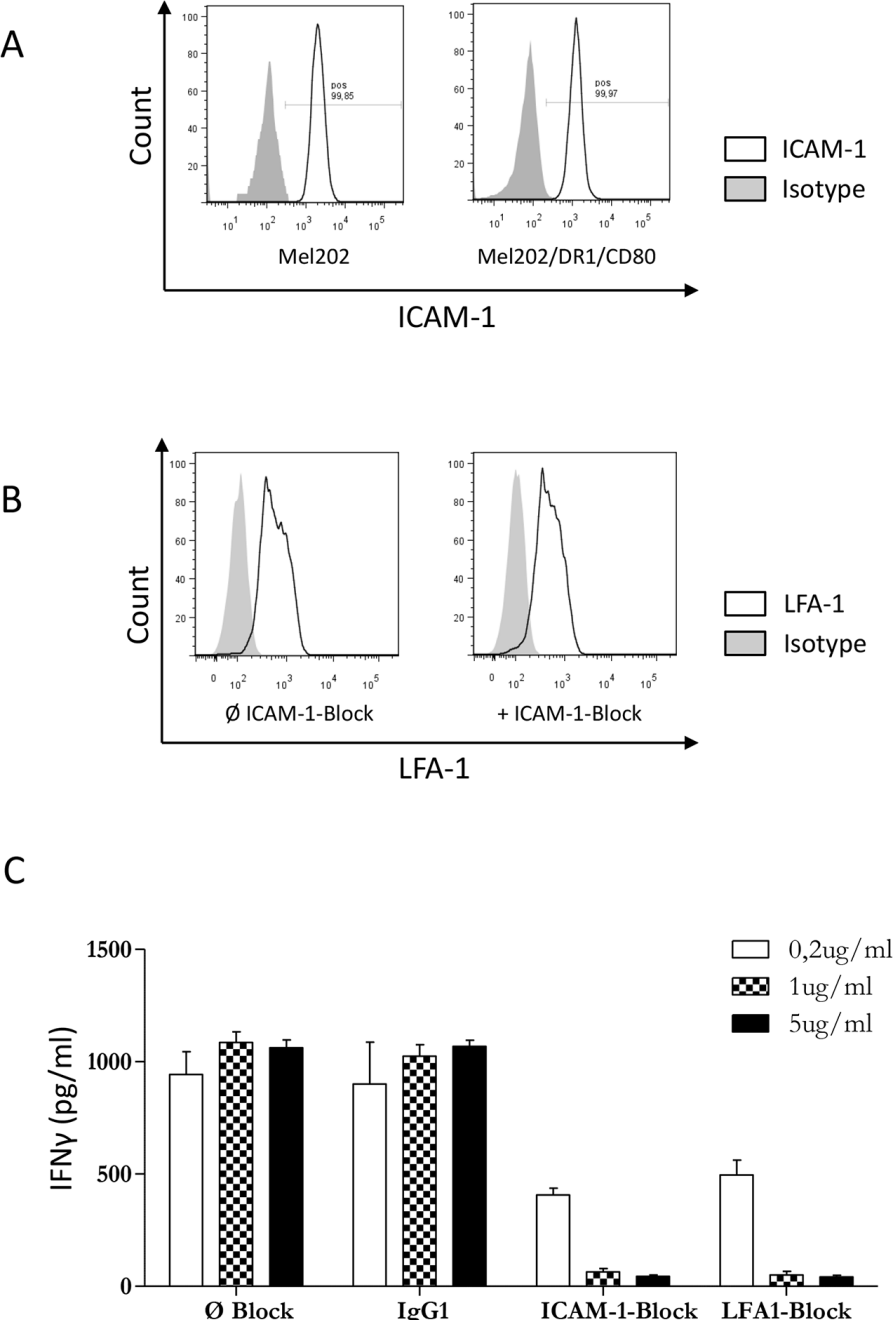
The ICAM-1/LFA-1 interaction is required for activation of CD4+ T cells by MHC II uveal melanoma vaccines **(A)** Mel202/DR1/CD80 and parental Mel202 cells express ICAM-1. Live uveal melanoma cells were stained for plasma membrane ICAM-1 (CD54). **(B)** Expression of LFA-1 by vaccine-activated CD4+ T cells in the presence (+) or absence (Ø) of ICAM-1 or IgG1 isotype control blocking antibody was analyzed after gating on CD3+/CD4+ T cells within the live gate. **(C)** Purified CD4+ T cells were primed and boosted with Mel202/DR1/CD80 in the presence or absence of ICAM-1, LFA-1 or IgG1 isotype control blocking antibody in a concentration range of 0.2-5μg/ml. T cell activation was quantified by measuring IFNγ-release. Data are representative of three independent experiments with CD4+ T cells from 3 individual donors.

In order to activate CD4+ T cells, we have previously shown that it is necessary for Mel202/DR1/CD80 vaccine cells to express MHC II and the costimulatory molecule CD80 [7]. However, cell-to-cell contact via adhesion molecules is a third component necessary for T cell activation. ICAM-1 is a pivotal intercellular adhesion molecule and a ligand for LFA-1, which is a prominent molecule of the integrin family of receptors expressed by leukocytes [30]. Since the role of adhesion molecules in activation of CD4+ T cells by MHC II vaccine cells is unknown, we tested the function of the ICAM-1/LFA-1 interaction. ICAM-1 is expressed by Mel202 wild type and Mel202/DR1/CD80 vaccine cells (Figure 9A) and vaccine-activated CD4+ T cells expressed LFA-1 that was unchanged in the presence or absence of ICAM-1 blockade (Figure 9B). Blocking of either ICAM-1 or LFA-1 resulted in a >10-fold decrease of IFNγ-secretion (Figure 9C). Therefore, the ICAM-1/LFA-1 interaction plays an important role in the activation of CD4+ T cells by Mel202/DR1/CD80 uveal melanoma vaccines. These data indicate that expression of cellular adhesion molecules in combination with antigen presentation and costimulation are required for successful activation of CD4+ T cells by MHC II uveal melanoma vaccines.

## DISCUSSION

The present data demonstrate that MHCII primary uveal melanoma vaccines directly prime and boost a diverse repertoire of purified CD4+ T cells. The CD4+ T cells secreted cytokines, proliferated and expressed a polyclonal TCR-Vβ profile with an activation phenotype. These findings extend the results of our previous uveal melanoma vaccine studies that used total PBMC. Since PBMC include other mononuclear blood cells, particularly professional antigen presenting cells, such as DC [7], it is important to demonstrate that the vaccines directly activate CD4+ T cells. By using magnetic bead isolated, highly purified CD4+ cells in our prime-boost experiments we could eliminate possible effects of accessory cells that might enhance T cell activation and show that CD4+ T cell activation occurs by direct contact with Mel202/DR1/CD80 vaccine cells. Furthermore, we showed that adhesion via ICAM-1/LFA-1 interactions facilitate the cell-to-cell contact required for direct CD4+ cell activation. The present studies in combination with earlier studies demonstrating that MHC II vaccines also activate CD4+ T cells by cross-dressing of DC [31], indicate that the MHC II vaccines may have enhanced T cell activation potential over other vaccination procedures, because they use two mechanisms for priming tumor-reactive T cells.

The activated CD4+ T cells proliferate and can be maintained *in vitro* for prolonged periods of time. These findings indicate that we have established a protocol that leads to the maintenance, priming and boosting as well as expansion of tumor-reactive CD4+ T cells *ex vivo*, a prerequisite of ACT with CD4+ T cells. Recent studies on melanoma and other solid tumors show the high potential of ACT. For ACT either peripheral T lymphocytes or tumor infiltrating T lymphocytes (TIL) are used. TILs have been obtained by isolation from tissue or biopsies of the primary or metastatic tumor and contain tumor-reactive T cells. In general, the challenge is to efficiently expand T lymphocytes *ex vivo* in order to obtain sufficient numbers for ACT. Many protocols use the non-specific expansion of TILs via CD3/CD28 bead activation in combination with cytokine support. There is considerable variability of responses between donors. Also in our present study, we observed donor variability in vaccine responses and TCR repertoire, which indicates some individuals are better responders than others.

In uveal melanoma, isolation of tumor cells for vaccine production and expansion of TILs for ACT is possible. Primary tumor tissue can be acquired via biopsy or resection of the tumor from the eye and used in vaccination protocols [32]. In addition, isolation, expansion and clinical use of TILs from metastatic tissue in the liver have been reported for uveal melanoma patients [33, 34]. Another way of gaining tumor-specific T cells is to expand them directly from the patient’s PBMC. For other malignancies it has been shown that this approach is possible by producing tumor-specific CTL via isolation of the patient’s PBMC and priming and boosting these T cells using tumor antigen loaded DC [35]. In the present study, we show that this strategy could be applied for PBMC in combination with MHC II uveal melanoma cell-based vaccines and cytokine support. The clinical use of whole tumor cell-based vaccines has drawbacks [36], however we favor this type of reagent for *ex vivo* applications, because these vaccines exploit the full scope of tumor (neo)antigens, thus potentially promoting broad tumor-specific T cell activation [37].

We focused on the activation of CD4+ cells, since there are several advantages of using tumor specific CD4+ T cells. CD4+ T cells are the orchestrators of the immune response and therefore they have great potential to recruit other cells such as CD8+ CTL or natural killer (NK) cells to the site of the tumor. We have previously demonstrated that uveal melanoma MHC II vaccines activate uveal melanoma-specific, cytolytic CD8+ T cells [8]. Therefore, ACT by CD4+ cells could complement vaccine-activated cytotoxic CD8+ T cells. The cytokines produced by vaccine-activated CD4+ T cells indicated that the CD4+ T cell response is diverse, contains Th1, Th2 and Th17 cells and that the population includes Tregs. In cancer Tregs are predominantly immune suppressive [38]. Nevertheless, the primed and boosted CD4+ T cells produced high amounts of the anti-tumor-cytokine IFNγ indicating that in spite of the presence of potentially immunosuppressive T cell subsets effective anti-tumor-activity is generated. Thus, this dual function of stimulatory and regulatory effects of IFNγ on anti-tumor immunity can be exploited by vaccine-activated CD4+ T cells [39]. In addition, we observed that repeated stimulation of CD4+ T cells with the vaccine cells seems to result in the accumulation of Tregs and secretion of IL-10. Although depletion of Tregs could enhance the CD4+ T cell response to the vaccine cells, Tregs might also improve anti-tumor immunity by promoting the generation of CD8+ T memory cells via production of IL-10 [40, 41]. Furthermore, the heterogeneous CD4+ T cell response corresponds with the diverse TCR repertoire and is indicative of a polyclonal CD4+ T cell response to multiple tumor antigens.

To improve overall survival in uveal melanoma it is essential to treat or prevent patients from developing metastatic disease. Patients with high metastatic risk can be identified using validated prognostic tests such as chromosome 3 status [42], gene testing for class 1 (low metastatic risk) and class 2 (high metastatic risk) [43] or molecular stratification according to somatic copy number alterations and DNA methylation profiles [44]. There are innovative clinical trials in which high risk, monosomy 3, HLA-A2+ uveal melanoma patients are vaccinated with either melanoma antigen-derived peptides or melanoma antigen-encoding mRNA transfected-DC in the prophylactic setting [45–48]. These immunotherapy trials are unique in that patients are vaccinated at early time points when there is no clinical evidence of metastatic disease. Furthermore, at this stage of the disease bulky metastatic tumor load is absent and patients are probably not immunosuppressed and hence more responsive to active immunotherapy. If these immunotherapeutic interventions are well tolerated and the results of these studies show therapeutic efficacy, another step has been made in establishing immunotherapy with tumor vaccines as a treatment option for uveal melanoma. This could make way for further studies exploring different approaches of immunotherapy such as vaccination in combination with ACT using tumor specific CD4+ T cells.

## MATERIALS AND METHODS

### Cell lines and peripheral blood mononuclear cells (PBMC)

Primary uveal melanoma cell line Mel202 was established from a uveal melanoma patient as described [49]. Mel202/DR1/CD80 vaccine cells were produced as described [7, 10]. Briefly, Mel202 cells were retrovirally transduced and selected to stably express HLA-DR1 and CD80. The vaccine HLA-DR1 genotype is HLA-DRB1*0101. Cell lines were cultured at 37°C, 5% CO_2_. Cells were grown in RPMI 1640 (ThermoFischer) with 1% L-Glutamin (ThermoFischer), 1% sodium pyruvate (PAN-Biotech, Germany), 1% MEM (PAN-Biotech), 0,4% MEM Vitamin Solution (choline chloride, folic acid, myo-inositol, niacinamide, D-pantothenic acid (hemicalcium), pyridoxal-HCL, riboflavin, thiamine-HCL, sodium chloride) (PAN-Biotech), 0,4% PenStrep (ThermoFischer), 0,1% mercaptoethanol (ThermoFischer) and 10% heat inactivated fetal bovine serum (FBS) (C-C-Pro, Germany). Blood samples and PBMC: Blood samples were obtained from healthy donors by leukapheresis. All cell lines and procedures with human materials were approved by the Institutional Review Boards of the participating institutions.

### CD4+ T cell isolation from PBMC

PBMC from HLA-DR1 positive donors (HLA-DRB1*0101) were obtained by leukapheresis, aliquoted and cryopreserved until usage. After thawing, CD4+ T cells were negatively selected using a MACS CD4+ T cell isolation kit II (Miltenyi Biotec), according to the manufacturer’s instructions. After isolation, CD4+ T cells were analyzed by flow cytometry and showed a viability and purity of >90%.

### T cell priming and boosting with Mel202/DR1/CD80 vaccines

Purified CD4+ T cells (2.5x10^6^) were mixed with irradiated (100 Gray (Gy)) Mel202/DR1/CD80 vaccine cells or Mel202 wild type cells (2.5x10^5^) or set up alone and incubated in 24-well plates at 37°C, 5%CO_2_. Cells were grown in T cell medium, which consists of RPMI 1640 (ThermoFischer) with 1% L-Glutamin (ThermoFischer), 1% sodium pyruvate (PAN-Biotech), 1% MEM (PAN-Biotech), 0,4% MEM Vitamin Solution (PAN-Biotech), 0,4% PenStrep (ThermoFischer), 0,1% mercaptoethanol (ThermoFischer) and 10% heat inactivated human AB Serum (PAN-Biotech).

After 2-3 days, “primed” T cells were harvested, washed, counted and replated for stimulation in 24-well plates with 10 ng/ml of interleukin 7 (IL-7) and 10 ng/ml IL-15 at 1x10^6^ cells/2 ml in T cell medium. On day 5-7, stimulated T cells were harvested, washed, counted and plated in 24-well plates without IL-7 and IL-15 and rested for another 1-2 days (1x10^6^ cells/2ml medium). Then, T cells were harvested, washed, counted and set up in 96-well U-bottom plates with Mel202/DR1/CD80 vaccine cells or Mel202 wild type cells at a ratio of 1:2 (5x10^5^ T cells and 2,5x10^5^ tumor cells) in triplicates and incubated at 37°C, 5% CO_2_. As controls T cells alone were used. After 48 hours, supernatant was collected and “boosted” cells were harvested for further analysis. For repetitive boosting, this sequence of cytokine support with IL-7 and IL-15, resting and boosting with Mel202/DR1/CD80 vaccine cells was repeated 2-8x.

### Cytokine analysis

Supernatants were assayed for IFNγ by ELISA using the OptEIA Human IFNγ ELISA Set (BD Biosciences). Supernatants were diluted 1:10 and stained and assayed as specified in the manufacturer’s instructions. Values are the averages of triplicate data points ± SEM. In addition, supernatants were analyzed for Th1 and Th2 cytokines using Human Th1/Th2 11plex Ready-to-Use FlowCytomix Multiplex (eBioscience) according to the manufacturer’s instructions. The following cytokines were analyzed: IL-1β, IL-2, IL-4, IL-5, IL-6, IL-8, IL-10, IL-12 (p70), IL-17A, TNFα, TNFβ and IFNγ.

### Flow cytometry

Monoclonal antibodies (mAbs) CD3-PerCP (clone SK7), CD4-V450 (Clone RPA-T4), CD25-APC-H7 (clone M-A251), HLA-DR-PE (clone L243), CD54-PE (ICAM-1; clone 84H19), CD11a-PE (LFA1; clone A2MR-α2), CD127, Ki67 and matched isotype controls were from BD Biosciences. All samples were run on a FACS Canto II Flow Cytometer (BD) and analyzed by FlowJo Software (Tree Star Inc., Ashland, USA).

### CFSE proliferation assay

5-8x10^6^ purified CD4+ T cells were labeled with carboxyfluorescein succinimidyl ester (CFSE) (Sigma) in a 5mM CFSE in phosphate buffered saline (PBS) solution for 4 min. T cell viability after CFSE labeling was >95%. CFSE-labeled CD4+ T cells were co-cultured with Mel202/DR1/CD80 vaccine cells or Mel202 wild type cells in 48-well flat-bottomed plates at a final volume of 1.0 ml per well in priming and boosting ratios as described above. As positive control, 5x10^5^ CFSE-labeled CD4^+^ T cells were incubated with 1, 5x10^5^ anti-CD3/CD28 Dynabeads® Human T Activator CD3/CD28 (ThermoFischer). After 48 hours, cells were collected, washed, stained for CD3 and CD4, and subsequently analyzed by flow cytometry as described above.

### TCR-Vβ phenotyping

The TCR-Vβ repertoire of vaccine-activated CD4+ T cells was determined using the IO Test Beta Mark TCR-Vβ Repertoire kit (Beckmann Coulter) according to the manufacturer’s instructions. This kit consists of mAbs to 24 distinct TCR-Vβ families (approximately 70% coverage of the normal human TCR-Vβ repertoire). CD3+CD4+ cells were stained and analyzed for TCR-Vβ usage according to the manufacturer's instructions.

### FoxP3 analysis

For real-time PCR (RT-PCR) of FoxP3 expression, mRNA was extracted from vaccine-activated CD4+ T cells using the peqGOLD Total RNA Kit (Peqlab). Extracted mRNA was reverse transcribed into cDNA (AffinityScript Multiple Temperature RT; Agilent Technologies). SYBR^®^ Select Master Mix (Life Technologies) and gene specific primers (QuantiTect Primer Assay; Qiagen) for human FoxP3 and HPRT were used for RT-PCR analysis. To compare relative FoxP3 expression between different probes, HPRT was used as reference gene and baseline day-0 cells served as reference probe (relative FoxP3 expression = 1).

For flow cytometric analysis of intracellular FoxP3 expression, cells were stained with anti-FoxP3-PE antibody (clone: 236A/E7; ebioscience) in combination with the FoxP3/Transcription Factor Staining Buffer Set (ebioscience) according to the manufacturer’s instructions.

### ICAM-1/LFA-1 blocking

Blocking antibodies against ICAM-1 (CD54) and LFA-1 (CD11a), and matched isotype control IgG_1_ (all BD Biosciences) were added to the CD4+ T cell/vaccine cell co-cultures at boosting in a concentration range of 0,2-5μg/ml. Mel202 and Mel202/DR1/CD80 cells were analyzed for ICAM-1 expression, and vaccine-activated CD4+ T cells were analyzed for LFA-1 expression by flow cytometry.

### Statistical analysis

SEM and 2-way anova were calculated using GraphPad Prism 5.03. For flow cytometry data analysis software programs FACS-DIVA (BD Biosciences, Heidelberg, Germany) or FlowJo (Tree Star Inc., Ashland, USA) were used.

## Abbreviations

ACT: adoptive cell transfer
CFSE: carboxyfluorescein succinimidyl ester
CTL: cytotoxic T lymphocyte
DC: dendritic cell
FoxP3: forkhead box protein P3
HLA: human leukocyte antigen
ICAM-1: intercellular adhesion molecule 1
IFNγ: interferon γ
IL: interleukin
LFA-1: lymphocyte function-associated antigen 1
MHC: major histocompatibility complex
NK: natural killer cell
PBMC: peripheral blood mononuclear cells
PD-L1: programmed-death-ligand 1
TCR: T cell receptor
Th: T helper type
TIL: tumor infiltrating lymphocyte
TNFα: tumor necrosis factor α
Treg: T regulatory cell
